# We are not doing enough: Truth-telling and Aboriginal and Torres Strait Islander history in Australian Public Health

**DOI:** 10.1371/journal.pgph.0004197

**Published:** 2025-03-12

**Authors:** Jasper Garay, Michelle Dickson, Candace Angelo, Karina Clarkson, Joel Dixon, Anthony Nicholls, Matilde Breth-Petersen, Diego S. Silva

**Affiliations:** 1 Aboriginal and Torres Strait Islander Public Health, Sydney School of Public Health, University of Sydney, Sydney, Australia; 2 Poche Centre for Indigenous Health, Faculty of Medicine and Health, University of Sydney, Sydney, Australia; 3 Sydney Health Ethics, Sydney School of Public Health, University of Sydney, Sydney, Australia; University of Greenwich, UNITED KINGDOM OF GREAT BRITAIN AND NORTHERN IRELAND

## Abstract

Most public health practitioners and researchers in Australia acknowledge the poorer health and wellbeing of Aboriginal and Torres Strait Islanders peoples relative to non-Indigenous Australians; some work with Aboriginal and Torres Strait Islander communities; however, few acknowledge the role that public health itself has played in the plight of Aboriginal and Torres Strait Islanders peoples throughout Australia’s colonial history. In this essay, we – Aboriginal, Torres Strait Islander, and non-Indigenous scholars at the Sydney School of Public Health (SSPH) – argue that truth-telling, which is critical for reconciliation, can only truly begin in Australian public health circles when we listen to the stories and truths of Aboriginal and Torres Strait Islander peoples as it relates to the colonial history of public health in Australia. Herein we give a brief outline of that history and provide some recommendations on ways forward; we also describe the successes and failures at SSPH in hopes that our story can help the broader Australian public health community on their truth-telling journeys. Although our story is about public health in Australia, we believe that its resonance lies everywhere public health has been explicit or implicit in its colonialism toward Indigenous peoples.

## Introduction

It’s beyond time we told the truth: as Australian public health researchers and practitioners, we aren’t doing enough to engage in truth-telling of how public health in Australia has impacted Aboriginal and Torres Strait Islander peoples. Engaging in truth-telling is essential if we are to do our part in supporting improved Aboriginal and Torres Strait Islander health and well-being. We are often quick to point out how others (e.g., politicians and governments) contribute to decision-making and actions that determine Aboriginal and Torres Strait Islander peoples’ ill health. However, Australian public health policies and practices have harmed and wronged Aboriginal and Torres Strait Islander peoples, too. 

Colonialism, imperialism, and racism informed the ideas and actions of many academics who grew the discipline of public health in Australia [1]. In the Australian context, colonisation sought to intentionally degrade, oppress, exclude, and manipulate Aboriginal and Torres Strait Islander peoples and society [2]. Public health’s success in this country was severely detrimental to Aboriginal and Torres Strait Islander peoples. While public health in Australia was implemented to support the acquisition and maintenance of good population health and wellbeing of White persons, it did very little to address the unjust realities forced upon Aboriginal and Torres Strait Islander peoples; therefore, public health is complicit in State violence by its collective inaction, if not actively prejudicial by the mid-20th century [3]. As public health researchers and practitioners, we don’t adequately acknowledge these wrongs or how they came about, and we don’t acknowledge their severity and the ongoing impact of that history on our present-day work. Today, the colonial foundations of public health take the form of inequalities in health and wellbeing outcomes [4]. We have taken insufficient responsibility for our wrongs and have not publicly apologised to Aboriginal and Torres Strait Islander individuals and communities for these wrongs. As it stands, we - individually, collectively, and institutionally - are not doing enough.

Truth-telling is a priority for Aboriginal and Torres Strait Islander individuals and communities, and is widely recognised as essential to healing, reconciliation, and health [5,6]. Public health prides itself in promoting and maintaining good health and wellbeing through evidence-based approaches. As such, we have an ethical obligation to listen to evidence consistently put forward by Aboriginal and Torres Strait Islander peoples, and to engage in truth-telling that describes the origins of Australian public health in order to facilitate healing and reconciliation. Public health investments towards supporting reconciliation are increasingly supportive of strengths-based, culturally safe, and practical approaches to reform [7]. While the contemporary Australian public health community has a heightened understanding of why this is a priority, the negative results of the 2023 Voice to Parliament Referendum (which we describe below) highlights that much work is yet to be done on the journey to achieving authentic reconciliation.

If the Australian public health community is not genuinely engaged in truth-telling, we will not progress a reconciliation that supports improved health and wellbeing for Aboriginal and Torres Strait Islander individuals and communities. Pathways leading to resolutions must begin where it all started, in our schools of public health. In this essay, we firstly seek to outline the need for historical truth-telling in Australian public health by beginning with a description of this history and ending with proposals for ways forward for the field. In between these two sections, we will briefly describe the efforts we have undertaken at the Sydney School of Public Health (SSPH) at the University of Sydney (USYD) to reckon with our institution’s role in this history, sharing our successes and failures, and identifying the essential work we must continue to do; we hope our story will be useful to others.

This essay reflects the work of Aboriginal, Torres Strait Islander, and non-Indigenous academics who are committed to advancing Aboriginal and Torres Strait Islander affairs in Australia and the SSPH. As we engaged with truth-telling processes in the SSPH, we recognised that, despite our best efforts, we were making inadequate progress. Collectively, we decided to share these experiences with the broader public health community. Aboriginal and Torres Strait Islander authors J.G. (Darkinjung, Ngarigo), M.D. (Darkinjung, Ngarigo), C.A. (Yuin), K.C. (Gamillaroi, Dunghutti), J.D. (Warumungu, Kaytej), A.N. (Aboriginal and Torres Strait Islander), and non-Indigenous author M.P. work in the SSPH Aboriginal and Torres Strait Islander Public Health team. Aboriginal author M.D. is the Director of the USYD Poche Centre for Indigenous Health. Non-Indigenous author D.S. is a Senior Lecturer in Bioethics in SSPH with public health ethics expertise.

We hope to provide critical viewpoints that support increased engagement with the cultural, professional, and organisational components involved in truth-telling processes. We believe that individual public health workers and their institutions must increase involvement in truth-telling processes. Although our focus is Sydney and Australia, we believe our story and arguments would apply everywhere Indigenous peoples have been subjected to colonialism and its present-day manifestations.

Before we continue, a brief note on the language we use: in present-day Australia, the preferred term used by community is ‘Aboriginal and Torres Strait Islander’. We acknowledge that naming conventions differ with different communities, e.g., whether the preferred term used is ‘Indigenous’ as is popular in Canada or ‘Native peoples’ in the USA. We default to using Aboriginal and Torres Strait Islander given we’re writing about the Australian context but will use the terms used by other communities outside of Australia accordingly. We also acknowledge that these naming conventions are not settled and subject to debate and evolution.

## Truth-telling: the process

Truth-telling is a process involving targeted activities that aim to acknowledge historical injustices, reconcile the ongoing impacts of colonisation, and promote the strengths and resilience of Aboriginal and Torres Strait Islander peoples and cultures [8]. Reconciliation, i.e., the strengthening of relationships between Aboriginal and Torres Strait Islander and non-Indigenous people, is a longstanding socio-political priority recognised by Aboriginal and Torres Strait Islander communities as something beneficial for all Australian people and society [9]. If truth-telling is a fundamental component of reconciliation, and leads to improved health and wellbeing, then it is necessary that Australian public health is genuinely engaging in this process.

In the context of Australian public health, truth-telling must begin with Aboriginal and Torres Strait Islander peoples; they are the primary stakeholders who tell their stories and truths, and decide how those truths should be delivered [10,11]. Our role in public health is to listen to these truths and devise ways to incorporate them into our daily professional and personal lives [11]. The intergenerational silence and inaction on this matter has haunted Australian public health for far too long; however, moving forward with truth-telling will still need time - it will be imperfect, incremental, and ongoing [12]. Truth-telling is a deeply interpersonal and cross-cultural process that should deliver genuine outcomes of reconciliation and healing for all Australians, but particularly Aboriginal and Torres Strait Islanders peoples.

Truth-telling should not be viewed as an academic exercise, but an opportunity to commit to processes that should profoundly shape how we engage in our public health education and research [13]. For public health academics, researchers, and practitioners, truth-telling should focus on interpersonal, community, and social outcomes [14], not on prioritising inward focused academic initiatives. Truth-telling, and changing our practice, requires investment of time that might not be currently valued in Western academic metrics, like successful grant applications. As public health academics, researchers, and practitioners we need to be okay with this and, in turn, universities must explicitly support their academics on this front.

Aboriginal and Torres Strait Islander people, communities, and organisations deserve to know that public health academic communities are serious about truth-telling. The broader public health community should become comfortable with an uneasiness in discussing certain issues required in truth-telling and being unable to guarantee short-term outcomes. Public health practitioners and researchers must relinquish control and power when truth-telling with Aboriginal and Torres Strait Islander individuals, communities and organisations. Importantly, we acknowledge this will inevitably create an increased cultural and professional load for Aboriginal and Torres Strait Islander individuals, communities and organisations, and advocate for this to be acknowledged and resourced properly, while their efforts recognised officially through commendations and career promotions. As we outline below, the opportunity to directly address historical trauma and ongoing injustice through increased collaboration with the various public health institutions (including but not limited to universities) in Australia will require a sincere funding commitment.

## Australian public health and colonialism

USYD and SSPH were complicit in colonialism, imperialism, and racism, while these effects resonate to the present-day. Complicity with these wrongs has provided great benefits to the institution. Public health, as practiced by faculty members of the USYD, was forcibly done unto Aboriginal and Torres Strait Islander society. The actions of faculty members of USYD sit alongside the action of other universities and their schools of public health in upholding racist and colonial values and histories, e.g., the work of public health researchers from the Universities of Adelaide, Melbourne, and Queensland work in establishing the suitability of White Europeans to live and replace Aboriginal peoples in the tropical parts of Australia [15]. Public health practitioners and researchers across Australia dismissed Aboriginal and Torres Strait Islander ways of knowing, being, and doing that supported positive cultural, health, and wellbeing outcomes as inferior to those of White persons [16]. Aboriginal and Torres Strait Islander peoples themselves were dismissed as inferior, and viewed as subjects that public health should investigate [17]. Public health did not care to consider supporting public health initiatives that aligned ethically and socio-culturally with the needs of Aboriginal and Torres Strait Islander peoples [18]. Inequitable social, cultural, political, economic, health and wellbeing disparities between Aboriginal and Torres Strait Islander and non-Indigenous peoples serve as stark reminders of what happens when public health ideas, initiatives, and systems are created without considering the needs of specific population groups. Specific examples of this can be seen in increasing rates of Aboriginal and Torres Strait Islander suicide, incarceration, and out of home care. [19–21].

Although others have told this history more fully [15,22–24], we note that we are the oldest school of public health in Australia, being founded in 1930. To begin, we must remember that the School of Public Health and Tropical Medicine (SPHTM), the first school of public health in Australia, was originally established by the Commonwealth (i.e., Australian Federal government) as part of the Department of Health [15,22]. It was introduced as a mechanism of colonialism through the Commonwealth and integrated into Australia by academics of the USYD. Until the 1950s, public health research that focussed on Aboriginal and Torres Strait Islander peoples, health, and wellbeing was minimal [25]. Harvey Sutton, inaugural Director of SPHTM, during the first three-decades of SPHTM, pursued limited research on mainland Australia. Instead, Sutton was heavily engaged in catering to the Commonwealth’s tropical medicine interests in the Pacific, aiming to support the administration of Australia’s colonial territories [24,26]. Through establishing public health in Australia as a discipline that progressed colonialism and the Commonwealth’s public health agenda, while simultaneously excluding Aboriginal and Torres Strait Islander peoples, we must acknowledge that public health supported the broader colonial system that contributed towards widespread oppression of Aboriginal and Torres Strait Islander peoples across the country, often by making them invisible to other Australians [27].

Colonialism was dominant in the thoughts and actions of Sutton and the SPHTM. For example, Sutton provided eugenics education and research at the USYD, advocating for ‘the guarding against degeneration of the race by greater numbers and proportions of ‘duds’ [1,26,28,29]. Sutton supported the concept and ideals of ‘White Australia’, one of the most detrimental colonial ideologies that embedded racialized oppression into Australian colonial society [30,31]. Although the ‘White Australia Policy’ primarily targeted the exclusion of non-White Asian migrants to Australia through the Immigration Restriction Act 1901, this vision for a colonised Australian society again demonstrates how key figures who established public health in Australia were racists, and how their racism maintained that Aboriginal and Torres Strait Islander peoples should not be present in Australia’s future [32]. Additionally, it demonstrates that if Aboriginal and Torres Strait Islander peoples were to be present, they would be managed through assimilation into colonial society and supported by eugenic practices that disrupted Aboriginal and Torres Strait Islander peoples, identity, and culture [1].

Critically for present-day public health, Sutton - through SPHTM and USYD - entrenched colonial influences into Australian public health that produced harmful and discriminatory events in future decades toward Aboriginal and Torres Strait Islander peoples. For example, Cecil Cook, a graduate of the Sydney Faculty of Medicine and staff member of SPHTM, became Chief Medical Officer and Chief Protector of Aborigines in the Northern Territory, administering assimilationist policies centred on ‘breeding out the colour’ of ‘half-castes’ through sterilisation [33–35]. Through the SPHTM, public health training was provided for all medical officers in Australia. This enabled generations of public health professionals to be informed by the ideals and ambitions of White Australia. As a discipline, public health therefore trained individuals through the lens of colonialism to create the public health structure for White Australia. Funding was acquired to conduct research that facilitated an agenda that opposed the interests of Aboriginal and Torres Strait Islander and non-White peoples, perpetuating public health evidence that legitimised the goals of colonialism through public health as a discipline. We need to better recognise that, fundamentally, the SPHTM and Australian public health contributed significantly to progressing colonialism, including toward Aboriginal and Torres Strait Islanders peoples.

These few examples provide a small insight into just some of the unfortunate origins of public health in Australia; yet there is so much we still don’t know, which requires the dedicated work of historians alongside dialoguing with Aboriginal and Torres Strait Islander elders and communities. We acknowledge that the USYD as institution, and some of its schools and faculties, are currently providing varying levels of commitment to engage in truth-telling and reconciliation more deeply [36]. We applaud and welcome these efforts. Despite these truths, the foundational leaders of public health in Australia did achieve outcomes that have benefited Australian society. It is not our intention or position to deny this. Still, it’s unclear how many of us who work in public health know the entirety of its full and accurate history, and it’s anecdotally obvious that many of us are unwilling to embed truth-telling and reconciliation as core business. Thus, we need collective commitment and action toward truth-telling.

## The nascent truth-telling at the University of Sydney School of Public Health

*SSPH and the lead-up to the 2023 Voice Referendum*: In 2017, under the auspices of Australia’s main political parties, a First Nations National Constitutional Convention was led by community leaders ‘to discuss and agree on an approach to constitutional reform to recognise Aboriginal and Torres Strait Islander peoples’ [37]. The primary outcome of that convention was the Uluru Statement from the Heart, which articulated – among other conclusions – that sovereignty of Aboriginal and Torres Strait Islander peoples was never ceded, the importance of strengthening truth-telling and reconciliation processes, and the need for a National Voice to Parliament and Makarrata Commission [38]. Central to the Uluru Statement was the concept of Makarrata, a Yolngu word meaning ‘a coming together after a struggle, facing the facts of wrongs and living again in peace’ [39–41]. It was proposed that through an established Makarrata Commission, truth-telling about Australian history would be prioritised, leading to enhanced opportunities for reconciliation, enabling progressive agreement-making between governments and Aboriginal and Torres Strait Islander peoples to take place. The Voice to Parliament was intended to be a mechanism to constitutionally enshrine Aboriginal and Torres Strait Islander political participation and leadership on matters that affected Aboriginal and Torres Strait Islander communities. The Voice to Parliament would not have had the power to make actual decisions on these matters. Rather, an enhanced strategic capacity to engage in practical and ongoing consultation and advisory work that aligned with community priorities would have been made possible [37]. To change the Australian constitution, a public referendum was necessary, which took place in October 2023.

Between 2021 – 2023, an Uluru Statement from The Heart working group was established at SSPH. The working group was constituted of Aboriginal and Torres Strait Islander and non-Indigenous academics and professional staff, alongside support from many other SSPH community members, who engaged in formal discussions to establish SSPH specific principles that supported truth-telling and the Voice Referendum. The establishment of this group was an important milestone for the SSPH community. For Aboriginal and Torres Strait Islander colleagues, opportunities were created to provide leadership and strategic direction on Referendum advocacy and community actions. This enabled our team’s public health work, academic skills, cultural knowledge, and positionality within the school’s community to be better understood, valued, and endorsed. For our non-Indigenous colleagues, this working group enabled space and time within academic contexts to formally engage in leadership relating to public health and Aboriginal and Torres Strait Islander affairs, thereby offering a concrete path of allyship. Stemming from public discussions arising from the working group, many other members of the SSPH community increased involvement with Aboriginal and Torres Strait Islander affairs. In 2023, 165 members of the SSPH community publicly endorsed the principles co-designed by the working group (see Table 1). During one of the most pivotal moments in recent history of Aboriginal and Torres Strait Islander affairs, this unprecedented support for action on truth-telling and self-determination is a milestone and source of pride.

**Table 1:** Sydney school of public health’s support for the voice to parliament.

The members of Sydney School of Public health who have signed this open letter below recognise: with utmost respect the innate dignity of all Aboriginal and Torres Strait Islander peoples. the unique and rich knowledges of Aboriginal and Torres Strait Islander peoples. that Aboriginal and Torres Strait Islander peoples’ connection with Country is pivotal to life, learning, culture, ethics, values, a sense of self identity, and a sense of community. that the health and wellbeing of Aboriginal and Torres Strait Islander peoples cannot be separate from social, political, economic, and cultural determinants of health and wellness. that our staff and students are visitors on the unceded lands where we teach, study, research, and live.
We commit to: learn about Australia’s past and current experiences of invasion and violence against Aboriginal and Torres Strait Islander peoples. creating the necessary space for listening and truth-telling. learning from our Aboriginal and Torres Strait Islander students and staff, about how to promote public health in ways that contribute to healing and sustaining Country. building meaningful relationships with Aboriginal and Torres Strait Islander peoples and communities that are grounded in equality. orienting public health education and research toward a strengths-based model that has decolonisation at its core.
We call on Commonwealth and State governments to: create a Makarrata commissioned by Aboriginal and Torres Strait Islander peoples, to engage in meaningful truth-telling. be guided by Aboriginal and Torres Strait Islander knowledges of public health, wellbeing and healthcare. commit to entrenching the diversity of Aboriginal and Torres Strait Islander voices in Parliament and within the Constitution.

*The subsequent pain from the results of the Voice to Parliament referendum*: However, the Voice to Parliament was rejected by 60% of the Australian public [42]. Results of the Voice Referendum were difficult for Aboriginal and Torres Strait Islander peoples throughout the country. Increased experiences of psychological distress, racism, social division, and re-traumatisation were reported during the Referendum period [43]. Aboriginal and non-Indigenous academics experienced what everyone in support of the Yes campaign felt through the confusing months that followed. Understanding the meaning behind the outcome and the impacts this would have on Aboriginal and Torres Strait Islander affairs was complex. Elders and leaders of the Yes campaign publicly requested that a week of mourning was respected [44], with no discussions of the outcome or future to be held.

At the same time, academic duties and the calendar year were coming to an end. It was after this period of mourning when we at SSPH reverted to inaction. When you read the listed commitments, you see individuals within our SSPH community that have agreed to action. At the time and still today, these are an ambitious set of commitments. We knew the public nature of the Voice Referendum was an ideal moment to generate increased awareness, involvement, and action on initiatives of importance to truth-telling and reconciliation. Now that Aboriginal and Torres Strait Islander affairs have again transitioned away from the general interest of the public, there has been a noticeable decrease in individual and community involvement towards acting upon commitments made.

*Moving forward at SSPH*: Individual academics wanting to support Aboriginal and Torres Strait Islander affairs must become accountable to consistent involvement with tangible results. This will require the humility to continue to learn with and from Aboriginal and Torres Strait Islander colleagues, and the courage to speak up and know that, as allies, we will make mistakes in our actions from which we will have to learn (e.g., saying something unintentionally ignorant or insensitive but not repeating the same mistake). The referendum represented a peak in public involvement with Aboriginal and Torres Strait Islander affairs. Despite increased positive involvement for the Yes campaign, negative perspectives and politics against public health progress for Aboriginal and Torres Strait Islander affairs also became more predominant [45]. Critical discussions must take place to understand these perceptions on processes that would support progress for public health, truth-telling, and reconciliation.

For truth-telling to occur, we need to have meaningful relationships with local Aboriginal and Torres Strait Islander individuals, communities and organisations [10]. Our SSPH community needs to reflect on where this missing piece of the puzzle has gone, or if we ever had it. For example, the neighbourhood of Redfern – long a traditional hub of Aboriginal pride and community activities – sits approximately two kilometres from the main USYD campus, yet it’s unclear this proximity has ever led to meaningful dialogue on matters of public health. Another accepted fact are the socio-political and cultural tensions which are barriers to creating these relationships: our SSPH community needs to formalise Aboriginal and Torres Strait Islander and non-Indigenous academic collaboration to internally generate a community engagement framework. Formalising Aboriginal and Torres Strait Islander governance structures at the school level would enable Aboriginal and Torres Strait Islander individuals, communities, and organisations to genuinely engage with representatives of the SSPH. If we are not honest with ourselves and begin to create meaningful relationships with local Aboriginal and Torres Strait Islander individuals, communities and organisations – the people that our wrongs have impacted upon most severely – our actions will quite simply imply that we’re not there to listen.

*Moving forward at the USYD:* Although truth-telling processes have commenced within the USYD, and we welcome and applaud these efforts, it remains unclear what this looks like and involves. Structural inaccessibility to institutional guidance on truth-telling creates misguided and ill-informed efforts across disciplines, schools, and faculties. Initiatives being discussed at higher levels are not currently discussed with smaller academic communities supporting preliminary engagement with truth-telling work. Involvement of Aboriginal and Torres Strait Islander individuals, communities and organisations have not yet been explained. It is time to open dialogue, show leadership through vulnerability, and publicly admit inter-personal, cross-cultural, real-world outcomes matter more than facilitating a perfect institutional truth-telling process. This open dialogue is necessary to academically incentivise, ethically develop, and practically promote ways for Aboriginal and Torres Strait Islander and non-Indigenous academics to become involved in truth-telling, reconciliation, and healing work. It is imperative that discipline and value specific communities voice their concern with the lack of open dialogue and community engagement. As wisely stated by Bhiamie Willamson post Referendum, ‘Within the disappointment of defeat there remain opportunities for those brave enough and willing to embrace it’ [46].

## Moving forward for Australian public health

When considering truth-telling processes underway elsewhere, USYD and Australian public health more broadly are behind the efforts of others [47]. However, this is not all bad news; we can benefit from leveraging outcomes and evidence that is widely available to support our own truth-telling processes.

Critically, and not surprisingly, there are at least two key elements for meaningful truth-telling in Australian public health: listening and dialoguing with Aboriginal and Torres Strait Islander communities and possessing the resources necessary to act upon lessons learnt in meaningful ways.

How can public health listen and dialogue with Aboriginal and Torres Strait Islander people? Here are some examples from which to draw on:

- Reconciliation Australia’s reconciliation framework - this framework acknowledges that truth-telling is essential to reconciliation, providing five-dimensions to work on: race relations; equality and equity; institutional integrity; unity; and historical acceptance [48].
- Application of these dimensions within Australian reconciliation and truth-telling processes can be identified in the Yoorrook Justice Commission of Victoria. Since 2022, the Yoorrook Justice Commission has held ‘public hearings’, allowing evidence from Aboriginal and Torres Strait Islander peoples, academics, descendants of colonisers, researchers, and historians to engage in truth-telling on injustices against Aboriginal and Torres Strait Islander peoples [49]. Truth-telling engagement in public hearings, by non-Indigenous Australians with family connections to controversial colonial histories, represents a significant step forward in non-Indigenous public involvement with Australian truth-telling and reconciliation.
- Another example is Melbourne University’s Dhoombak-Goobgoowana (truth-telling, Woi Wurrung language of the Wurundjeri Woi Wurrung people): among many colonial injustices, this truth-telling process has begun to examine the role of eugenics and medical research that impacted on Aboriginal and Torres Strait Islander peoples [50]. Aboriginal Elders, communities, academics, and organisations have been involved to ensure the process is facilitated through culturally safe and meaningful activities of benefit to Aboriginal peoples. In the recently published *A History of Indigenous Australia and the University of Melbourne: Volume 1,* Professor Marcia Langton reinforces the need to transcend academic reporting on historical evidence and outcomes, concluding that ‘Not only must we talk, we must also help to overcome the often-traumatic historical legacies and contribute to initiatives that redress the wrongs with respect and with regard to the rights of Indigenous peoples, as they are now elaborated in the United Nations Declaration on the Rights of Indigenous Peoples’ [50].
- In the United Kingdom, Cambridge University conducted a two-year inquiry examining historical links to colonialism, resulting in The Legacies of Enslavement report [51]. In support of the report’s recommendations, £1.5 million was invested towards research, community engagement and partnership activities through the Cambridge Legacies of Enslavement Fund.
- Harvard University accepted the recommendations as outlined in the *Report of the Presidential Committee on Harvard & the Legacy of Slavery* [52]*.* $100 million USD was committed to support implementation. In 2023, Harvard University’s Native American Program collaborated with the Harvard Radcliffe Institute to facilitate the conference ‘Responsibility and Repair’. Leveraging findings from the report, the conference bought Native peoples and university leaders to advance national dialogue, expand research, and enhance partnerships with Indigenous communities [53].
- In Canada, multiple universities have responded to the Truth and Reconciliation Commission of Canada: Calls To Action with institutional investment [54]. The National Centre for Truth and Reconciliation (NCTR), at The University of Manitoba, serves as the permanent archive for materials and records. Truth-telling is core business for the NCTR, working with Indigenous and non-Indigenous peoples to ‘honour Survivors and to foster reconciliation and healing on the foundation of truth-telling’ [55]. The Indian Residential School History and Dialogue Centre, at the University of British Columbia, addresses colonial impacts on Indigenous peoples inflicted through the Canadian government. One key goal is to ensure Residential School Survivors, and their families, are supported to access historical records and documentation, enabling healing and reconciliation through enhanced capacity for Indigenous peoples to engage in truth-telling [56].

These examples of truth-telling processes demonstrate varied, yet meaningful, approaches that seek reconciliation by addressing the reality of impacts stemming from colonialism, imperialism, and racism. They’re likely imperfect, but meaningful first efforts. Indigenous peoples and communities have led in truth-telling activities, gained increased access to information that supports lived experiences and testimonies of historical injustices, and importantly, have further embarked on journeys towards healing and reconciliation. Institutions have made financial investments towards reforms of benefit to communities affected, reinforced the need for non-Indigenous accountability and engagement with reconciliation, and committed to continued dialogue and actions that will enable more equitable relations between colonial society and Indigenous communities. In the Australian public health context, we are well positioned to be guided from this evidence to generate meaningful outcomes from our own truth-telling processes.

## Conclusion

As an academic, research, and practice community, we recognise the importance of engaging in practice, research, and education that attempts to reconcile with these wrongs. We acknowledge there are many well-respected people and strengths-based initiatives doing good in Australian public health. However, we must ask individual public health researchers and practitioners:

1. Are you genuinely contributing to correcting these wrongs through your academic work?
2. Are we genuinely doing enough to engage in Aboriginal and Torres Strait Islander community focussed, meaningful, and practical truth-telling?
3. Are we genuinely thinking about what actions will be required from the Australian public health community to support progressive outcomes arising from the truth-telling process?
4. Are we genuinely comfortable with the state of Australian public health considering our objective inaction on reconciling with colonialism, imperialism, and racism?
